# ISCB Student Council Symposium 2021, a virtual global venue: challenges and lessons learned

**DOI:** 10.12688/f1000research.129945.1

**Published:** 2023-01-12

**Authors:** Cleidy Osorio-Mogollon, Victor Grentzinger, Gabriel J. Olguin-Orellana, Sebastian Ayala-Ruano, Shruti Gupta, Pradeep Eranti, Aayush Grover, Bart Cuypers, Nazeefa Fatima, Sayane Shome, Farzana Rahman, R. Gonzalo Parra

**Affiliations:** 1 Ribeirao Preto Medical School, University of Sao Paulo, Riberao Preto, Brazil; 2 Laboratory of Human Genetics, GIGA Research Institute, 4000 Liège, Belgium; 3 Center for Bioinformatics, Simulation and Modeling (CBSM), Faculty of Engineering, Universidad de Talca, Talca, Chile; 4 Faculty of Science and Engineering, Maastricht University, Maastricht, The Netherlands; 5 School of Computational and Integrative Sciences, Jawaharlal Nehru University, New Dehli, India; 6 Universite Paris Cit ´e, Inserm, UMRS-1124, Group of Genomic Epidemiology and Multifactorial Diseases, Paris, France; 7 International Institute of Information Technology Bangalore, Bangalore, India; 8 Adrem Data Lab, Department of Computer Science, University of Antwerp, Antwerp, Belgium; 9 Nordic Computational Biology, NA, Sweden; 10 Department of Anesthesiology, Perioperative, and Pain Medicine, Stanford University,, California, USA; 11 Faculty of Engineering, Computing and Environment, Kingston University, London, UK; 12 Computational Biology Group, Life Sciences Department, Barcelona Supercomputing Center, Barcelona, Spain

**Keywords:** student council, bioinformatics, computational biology, covid19, virtual, pandemic

## Abstract

Since 2004, the ISCB Student Council has been organizing different symposia worldwide, gathering together the community of young computational biologists. Due to the coronavirus disease 2019 (COVID-19) pandemic situation, the world scientific community was forced to cancel in-person meetings for almost two years, imposing the adoption of virtual formats instead. After the successful editions of our continental symposia in 2020 in the USA, Latin America, and Europe, we organized our flagship global event, the Student Council Symposium (SCS) 2021, trying to apply all previous lessons learned and to exploit the advantages that virtuality has to offer.

## Introduction

The International Society of Computational Biology - Student Council (ISCB-SC) is an international, global organization for students pursuing computational biology and bioinformatics. ISCB-SC focuses on promoting interaction among students and young researchers. To this extent, ISCB-SC organizes its largest event, held every other year, called Student Council Symposium (SCS). ^1^^,^^2^ This event allows students from around the globe to showcase their works in front of an international audience. Due to the pandemic, virtual events have been the solution for carrying out scientific dissemination events and networking. SCS2021 was no exception and was held on the Airmeet platform.

## Attendees

**Figure 1.**
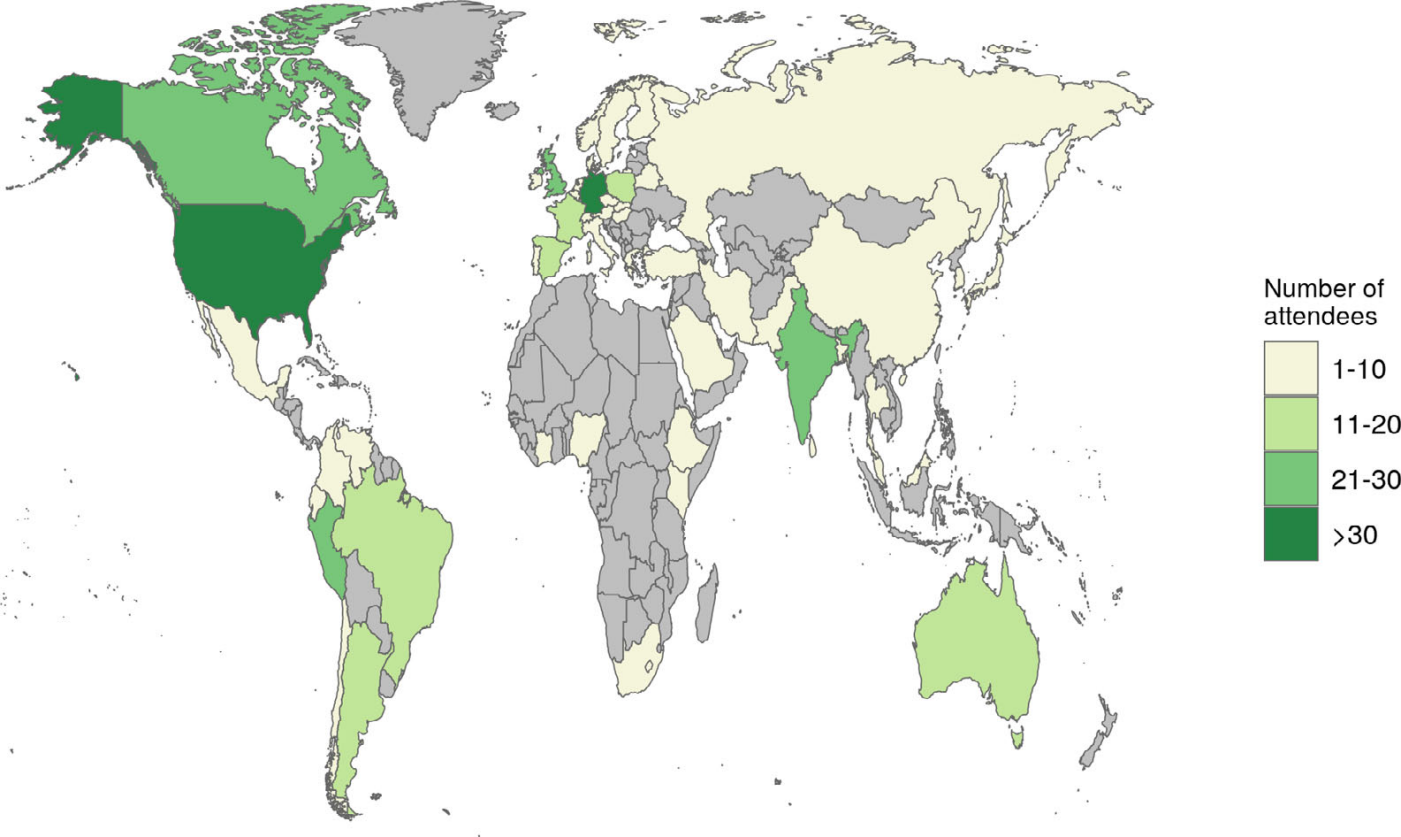
World map of registration to the SCS2021. This Figure was created with the ggplot2 R package. ^3^

We had 482 people registered for our event through the ISMB/ECCB 2021 website [ 1]. On the platform, 233 attendees connected at some time during the event, 40% of them joined a discussion table, and approximately a maximum of 95 people connected simultaneously in Airmeet. The demographics of attendees can be found in Figure 1.

We found out that people tend not to attend all talks but some of them. This could be the consequence of the virtual symposium, as people prefer to attend specific talks and not the entire program or people doing other activities in parallel, as a consequence of not being in a physical venue. Additionally, the free-to-attend nature of the meeting makes it easy for people to register without a strong incentive to maintain the attendance commitment. We should delineate strategies to encourage people to attend more talks. One strategy could be checking who attends which talk and giving prizes to those people who attend the most talks or who asked the best question.

## Virtual platforms

We consider a virtual platform with the following characteristics: rooms for the main conference and spaces for poster presentations, both are very important for the attendee’s presentation types (talk, flash talk, and posters); boxes for Q&A and chats for the interaction between the organizing committee and attendees and between attendees; and pool questions for the social event.

As the previous editions of symposiums organized by the Student Council ISCB, ^4^^,^^5^ Airmeet was selected as the main platform for the SCS2021. The advantages of using this platform were the variety of options that could be used for the smooth running of the event. For example, using tables (for a maximum of eight participants), with audio and video, for networking time. This Airmeet feature is very useful for closer interaction between participants and becomes unavailable when the conference starts so that attendees can pay attention to each presentation. Another advantage is that, before the event, the platform allows you to upload pre-recorded videos to be able to play them at the event. This strategy of playing videos was used to avoid possible connection problems at the time of the presentations of speakers. During conference time, questions and comments from attendees appear on the shared screen and are available to everyone.

The disadvantage of using the platform is the way of connection. Airmeet only allows full use of all its tools in one desktop application. Also, Airmeet has a restriction on the browser, which is preferably Chrome. These indications were notified to the attendees before the event. Finally, a very stable internet connection is needed to load the platform and this can be an issue for many people that are located in geographical regions where the internet connection is not good enough.

## Organization across time zones

The COVID-19 pandemic forced people to stay in their homes and led scientific communities to embrace the value of virtual events that can be attended by people from different corners of the world. With all the pros, the time-zone hurdle initially seems like a minor obstacle; however, it represents a significant part of the logistics associated with the organization of international all-day events and comes with its own set of challenges. Besides joining events spread across odd hours, managing proper sleep without disturbing the daily routine was challenging for the attendees, presenters, and organizers. Hence, the organization’s most challenge is fixing the events’ timing. This problem was less evident in the events organized by the Student Council in 2020 as they were continental and not global, as SCS2021.

To be respectful and inclusive of all our participants’ time zones, we split the one-day format adopted by earlier symposia into a two-day virtual event in 2020 and 2021, each day schedule spreading for 5 to 7 hours with sufficient breaks. Additionally, in 2021, we introduced different starting times for each day with a difference of 6 hours. We consider it to be more inclusive for participants and eased the organizers’ stress. Considering that the majority of participants are young researchers, we decided to schedule the talks for presenters at convenient times as per their UTC offsets instead of themed sessions. It enabled the participants to have more interactive and broadened Q&A sessions. We also structured the tasks among the organizers to fit across time zones (Based on local times of countries such as Peru, France, India, Argentina, the USA, México, Spain, and Germany). An essential and gripping part of the event, Keynote talks, needed to be scheduled to maximize participation. The three keynote talks at SCS2021 were spread over two days. An extended poster-panel session and networking were scheduled on both days to allow the participants to network among themselves and have a one-to-one live Q&A with presenters of posters and talks. The above considerations helped us to make our second virtual symposium a global and insightful experience.

## Social networks

For some years, social networks like Facebook, Twitter, and Instagram have become fundamental for scientific communities to reach their members as well as potential new target audiences. Due to the pandemic and the shift from in-person meetings to virtual ones, this tendency has deepened, becoming almost mandatory to ensure the successful organization of scientific events. As in the past editions of the ISCB-SC symposia, the designated SCS Outreach Committee has played a crucial role in the successful accomplishment of the SCS2021, making strategic use of its two subcommittees, Design and Social Networks, that worked in cooperation with all other committees, providing them with supplementary material and creating online posts to reach the maximum possible audience with one main motive: “ *If it does not exist online, it does not exist at all.*”

On the one hand, the Design subcommittee started its work by creating the graphic pieces used on the website, which implied the development of an artistic concept that reflects the event’s intention. For this year, two main ideas were put in the design line: the lore and traditions of France, the virtual venue of the SCS2021, expressed through the brush stains and the calligraphic typography, and the notion of innovation coming from computational biology through the squares that represent the computation and digitally. These concepts were then exported to other graphics, such as invitation letters, sponsorship packages, designs for social networks, and diplomas.

On the other hand, the Social Media subcommittee was in charge of attracting potential attendees and informing them about the event’s details using our digital platforms for this purpose. This year, we used five main communication channels: An email chain, a space in Basecamp for the ISCB-SC members, and three social network accounts especially dedicated to this symposium, i.e., Facebook, Instagram, and Twitter. Thereby, the email chain and the Basecamp space focused on contacting the students and researchers of the 15 different Regional Student Groups (RSGs) and other members within the ISCB-SC leadership. At the same time, Facebook, Instagram, and Twitter were used to recruit those people that had already participated in the past edition of the symposium and new ones interested in attending. Regarding the origin of these accounts, in the case of Facebook and Twitter, we used the ones created to promote previous editions of this symposium. This strategy of handing the same account in each new edition has been used for the four major symposia ISCB-SC (SCS, LA-SCS, E-SCS, and SCS-A), allowing us to start each new diffusion round in close contact with an audience already interested in the activities offered by the ISCB-SC. Also, it settled the structure for the social networks for the upcoming Symposia which will start already with 576 followers on Twitter (SCS2021), 3941 on Facebook (ISCB Student Council), and 178 on Instagram (SCS2021). In the case of the Instagram account, because we realized the fast increment of young people interested in learning bioinformatics due to its relevance in the development of solutions for the pandemic, for the first time, we created an Instagram account for the first time to attract them to our events. Although there was a long latency until we gained some followers, it will also facilitate the tasks for the next Symposia. Another advantage of using the same accounts for each new instance of this activity is the possibility of remaining in touch with the audience in the times between the events to continue sharing interesting information about computational biology, our institutions, and their activities and incentivizing people to engage in online discussions after the event.

The last communication platform used to share the results of the SCS2021 is YouTube, in which all the oral presentations, including the keynote and fast talk presentations, are available. This has been done in the ISCB account, with the ISMB/ECCB 2021 presentations, but in a dedicated playlist to our activity. In order to be respectful to the presenters, they are asked to give informed consent before uploading the videos.

## Keynote speakers

For this edition, we welcomed three keynote speakers: Prof. Wolfgang Huber from EMBL, whose topic was “Doing great (in) computational biology."; Prof. Nicola Mulder from the University of Cape Town, whose keynote was “Building capacity and resources to enable genomic medicine in Africa."; and Prof. Yana Bromberg from Rutgers University, who spoke about “Decoding the DNA blueprints of molecular functionality."

## Prof. Wolfgang Huber: Doing great (in) computational biology

**Figure 2.**
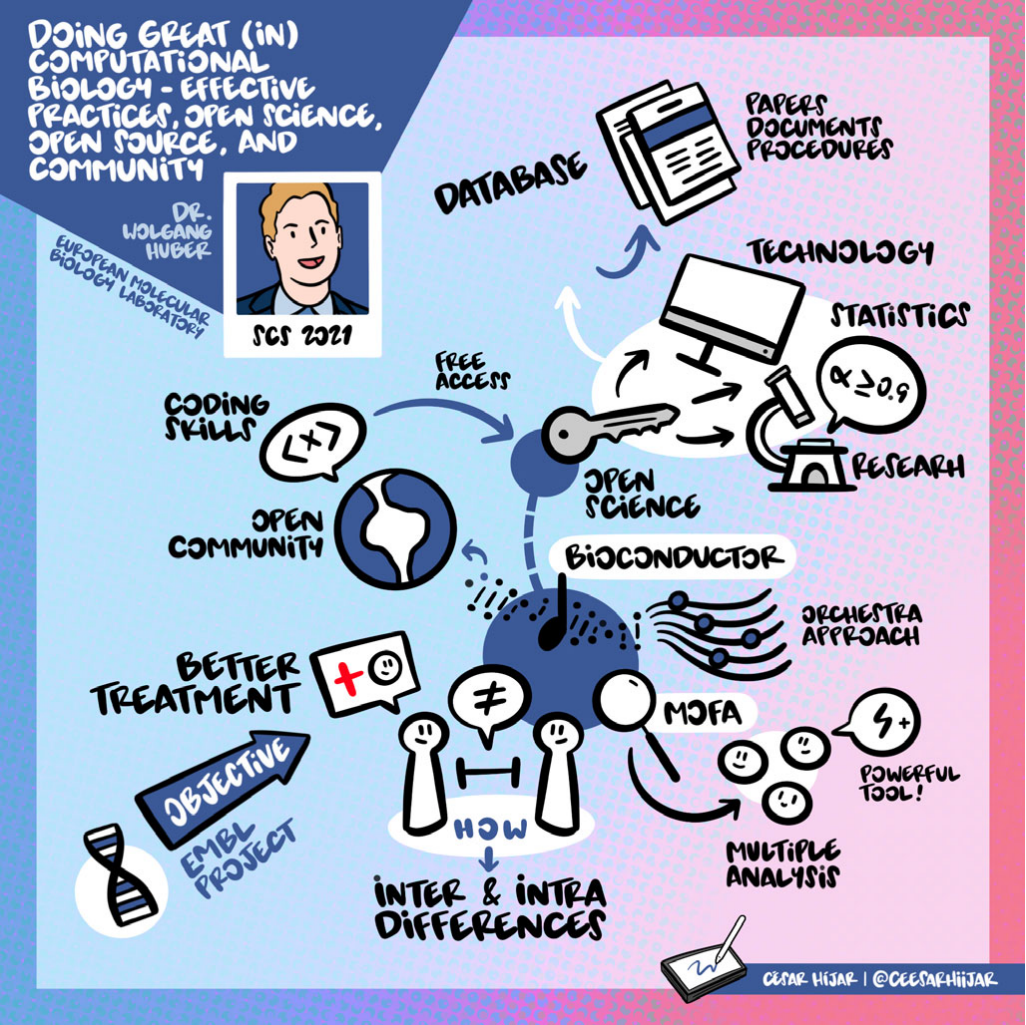
Summary of Prof. Huber’s talk. ^7^

Prof. Huber’s talk focussed on Open science and why it is an essential part of great computational biology projects. He explained how transparent and accessible research has better reproducibility, transparency, credibility, and usefulness. It also supports research in developing countries and cities. He also talked about steps taken at EMBL to make tools reusable, peer-reviewable, transparent, and access to FAIR data (findable, accessible, interoperable, and reusable). ^6^ For example, he discussed a collaborative software development project, Bioconductor, an R-based open tool with a support site, a repository, and an active community that continues to develop this software. He also emphasized that similar criteria should assess scientific software and a scientific publication. He advised the young researchers not to reinvent the wheel but reuse codes and do research with modularity, as monolithic research is not outstanding. The visual summary of his talk can be found in Figure 2.

## Prof. Nicola Mulder: Building capacity and resources to enable genomic medicine in Africa

**Figure 3.**
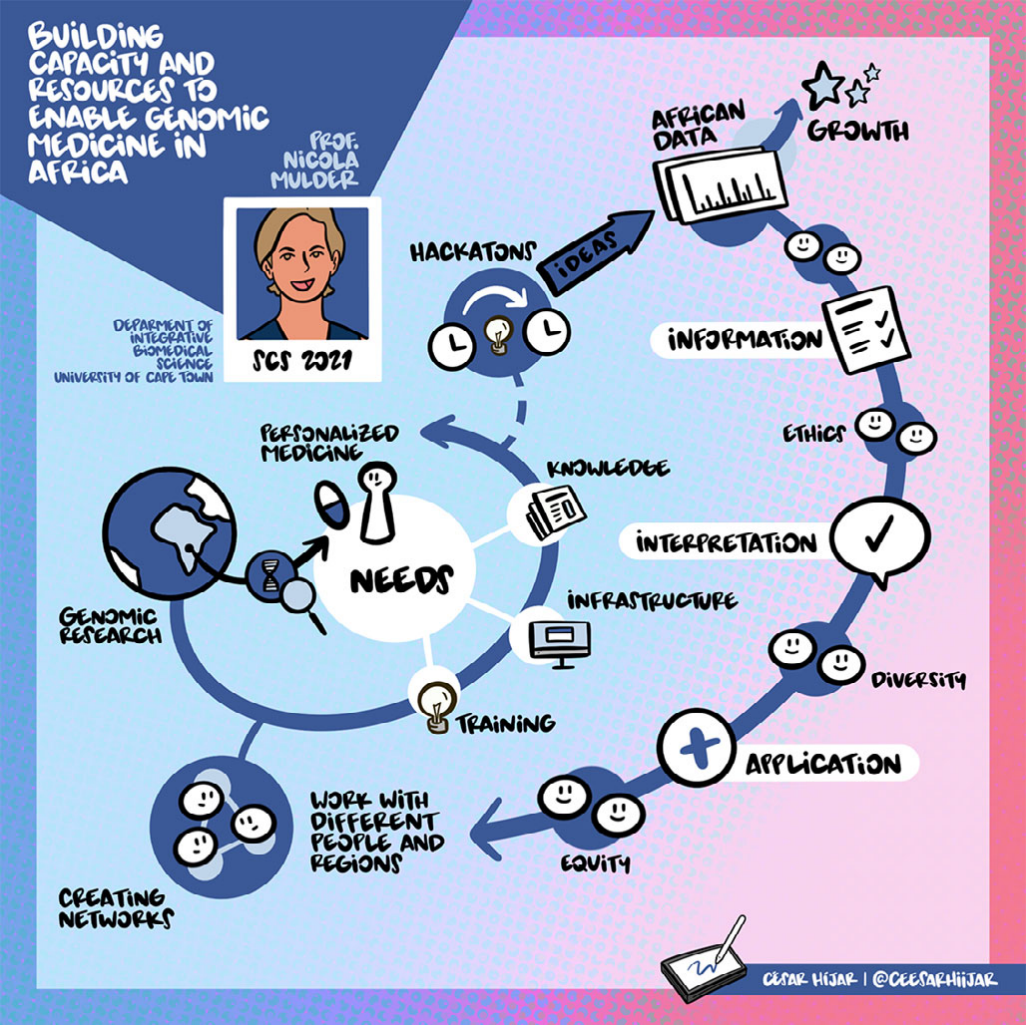
Summary of Prof. Mulder’s talk. ^7^

The standard approach to treating a disease is to use a single treatment for all. Prof. Mulder’s talk focused on how this approach is not applied in the African community and how it is designed for genomics medicine for the African community. In the case of Africa, a large number of diseases are present, and non-African genotypes-phenotypes associations cannot be effectively applied to African patients. Therefore, tailored African genomic medicine is important and should be developed. The challenges for African biomedical data are to have quality representation in reference genomes and public databases, as well as the protection of the rights and privacy of participants. In response, Human Heredity & Health in Africa (H3Africa) was born. This structure recovers and analyzes phenotypic, genomic, and microbiome data. The bioinformatics component is managed by the H3ABioNet branch, whose Principal Investigator is Prof. Mulder, enabling storage, analysis, and curation infrastructures. In addition, data analysis is done according to FAIR principles. This structure has enabled the surveillance of severe acute respiratory syndrome coronavirus 2 (SARS-CoV-2) and the tracking of variants, but also the development of tools for genomic medicine, phenotype harmonization, and analysis of African genome data. Figure 3 highlights the visual summary of her talk.

## Prof. Yana Bromberg: Decoding the DNA blueprints of molecular functionality

**Figure 4.**
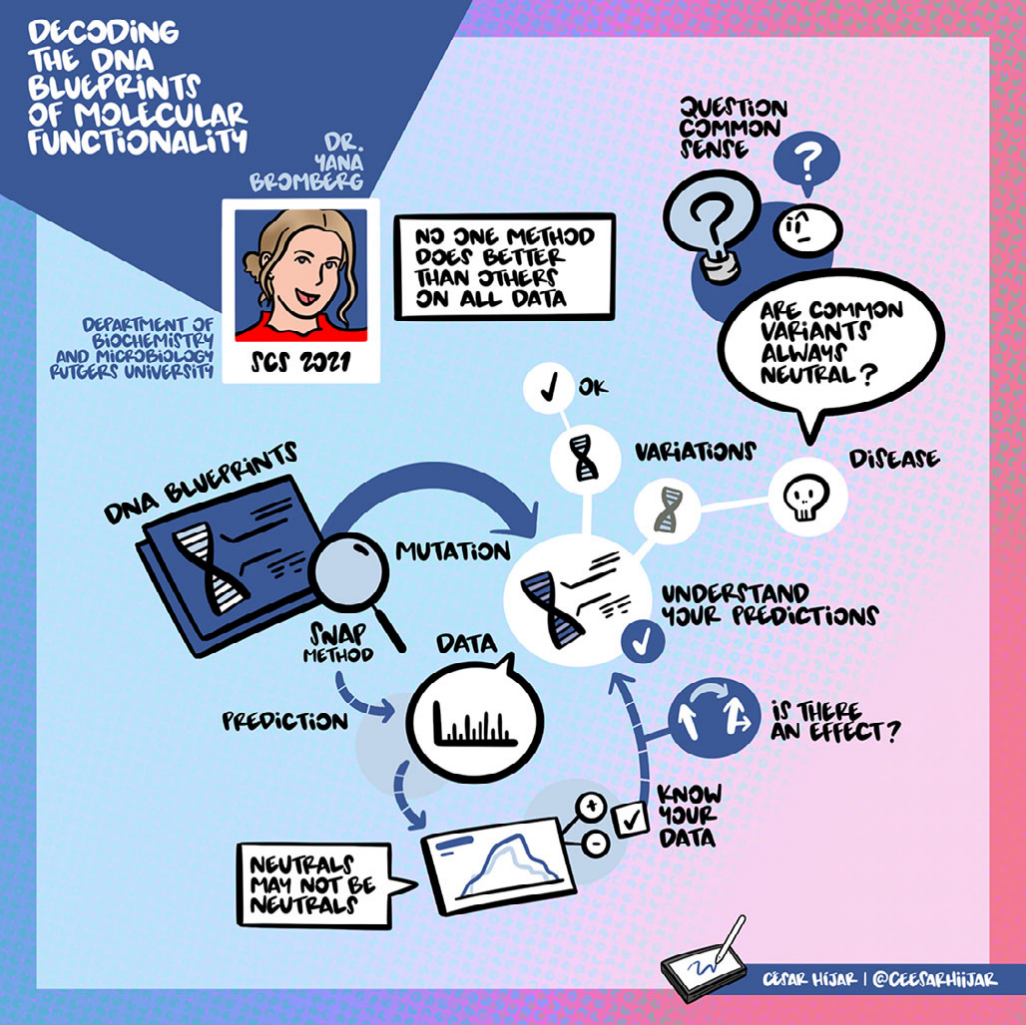
Summary of Prof. Bromberg’s talk. ^7^

Data is the backbone of any analysis. Prof. Bromberg emphasizes the importance of understanding the data and the predictions in any analysis. Especially in bioinformatics, it is extremely important to understand the biological significance of any prediction. Further, Prof. Bromberg also focuses on the significance of questioning “common sense.” She shows this in her work, where she uses the counterintuitive approach to understanding the biological impact of Synonymous Single Nucleotide Variants (sSNV). Her group observed that artificially generated exomes with sSNV play an equal role in the prediction of variant effects as compared to observed variants. They also show that using a combination of generated and observed variants helps improve the identification of function effects of variants. Finally, Prof. Bromberg recommended that researchers consider the background when looking for pathogenic variants. The visual summary of her talk can be found in Figure 4.

## Student and early-career researchers’ presentations

There were 12 oral presentations, 12 flash talks, and 24 posters for the live session. For each oral and flash talk presentation, authors were asked to submit a pre-recorded talk so that there were no delays and technical difficulties. However, we asked the speakers to be available for a live Q&A. For each poster, one of the authors was asked to be available to enable discussions with the audience. Eight reviewers selected these talks and presentations based on the quality and impact of their submissions.

**Figure 5.**
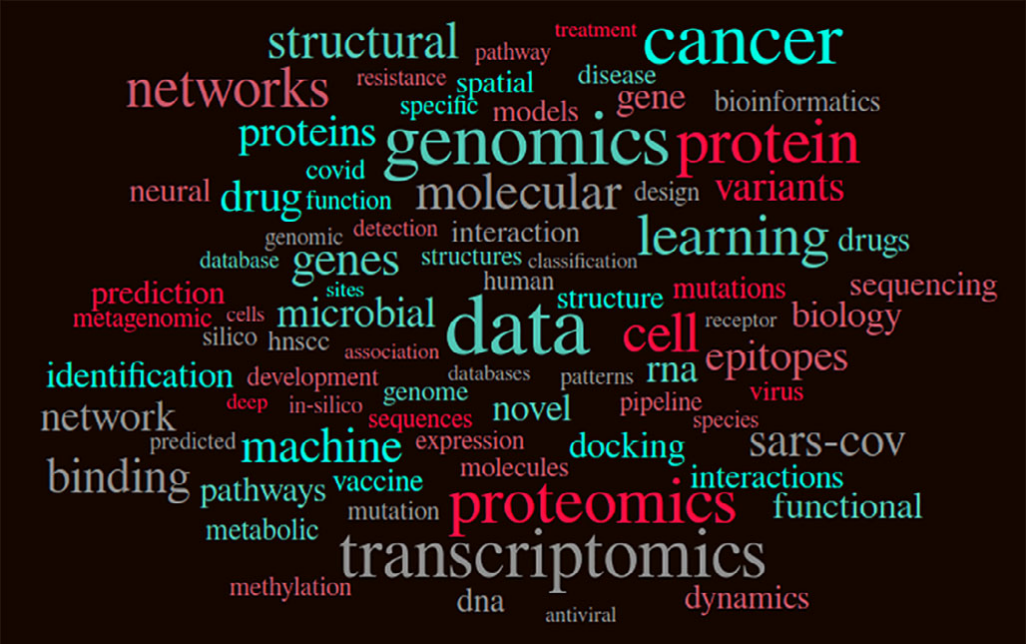
Word cloud made using the abstract text from all presented talks.

**Figure 6.**
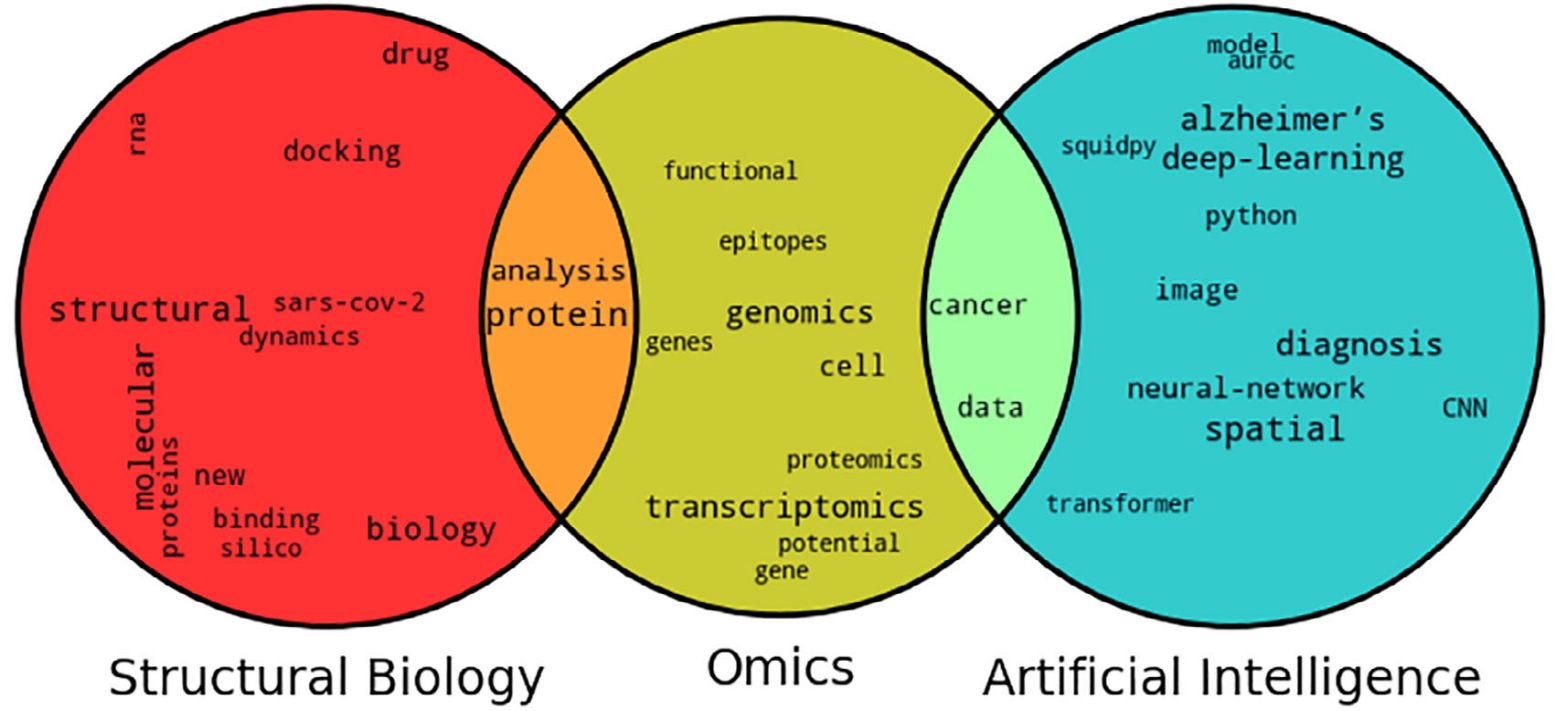
Venn diagram of word clouds based on submitted abstracts in each topic.

Over the two days, the talks and posters covered a plethora of biological topics - single-cell, cancer genomics, and drug repositioning, to name a few. The opportunity was also given to benchmarking works as they play an influential role in the field of research. Out of the twenty-four (24) oral and flash talks, we also witnessed three (3) talks on understanding and tackling COVID-19. A word cloud summarizing the abstracts for all talks is shown in Figure 5. We can broadly classify the topics of the talks into Structural Biology, Omics, and Artificial Intelligence. Bioinformatics and computational biology are highly multidisciplinary fields, we can see the significant overlap between these different topics. The Venn diagram of word clouds ( Figure 6) is formed based on the word frequency of the top 10 words used in the abstract of each work belonging to this topic category. It can be inferred that few omic studies focused on cancer as well as data, whereas several studies analyzed proteomics data for structural biology.

Through these multi-disciplinary talks, SCS2021 enjoyed discussions on various topics, allowing everyone from different subfields to teach and learn something new.

## 1st world wide RSG round table at SCS

The primary mission of the International Society for Computational Biology Student Council (ISCB-SC) is to nurture a new generation of computational biologists. Being part of the ISCB-SC, Regional Student Groups (RSGs) have provided a platform for young computational biologists and bioinformaticians to further their careers through knowledge dissemination and networking opportunities. ^8^ RSGs provide these networking opportunities locally in their respective countries, and regions, and internationally through the ISCB-SC events like the student council symposia. First of its kind, we have introduced the Global Leadership Round Table session to provide a unique networking opportunity, which was open to SCS participants and all the RSG members of the RSGs around the world, even without registration.

In normal, pre-pandemic times, and due to the high-associated costs of international traveling, a small fraction of the SC leadership was present at the previous in-person SC events, despite the worldwide presence of the RSGs. We have issued an open call to all the SC members worldwide to nominate leaders from their RSGs to share their achievements and possibilities in front of our global community. The Student Council’s Executive Team selected the representations from six RSGs: RSG-France, RSG-Spain, RSG-Chile, RSG-India, RSG-Bangladesh, and RSG-Turkey, covering both mature and newly created RSGs across Latin America, Asia, and Europe. The round table included presentations from the RSG committee chair and the leaders of six RSGs.

RSGs actively disseminate knowledge through webinar series, journal clubs, round tables, and involving undergraduate students and senior researchers. Furthermore, these early-career researchers-led groups organize tutorials ranging from general bioinformatics topics to focused ones such as Population genetics analysis and Single-cell data and workshops for scientific data visualization. RSGs actively do science communication through newsletters and Fête de la science (RSG-France), blog posts (RSG-Turkey), and/or Twitter experience sharing (RSG-Spain). RSG-Spain actively participates in the Bioinfo4Women [ 2] initiative and actively encourages the career progress of female students and researchers, especially those that are transitioning from the postdoc level to independent positions. RSG-Bangladesh encourages its volunteers to present at internal platforms such as the Student council symposia and SC webinar series program and is actively growing in the region.

RSG-Spain and RSG-Turkey have organized annual student symposia for a decade by actively liaising with the national bioinformatics associations and representing the students’ interests. ^9^ RSG-Chile has started in this direction and recently organized its first national symposium. RSG-Chile, RSG-India, and RSG-Spain are expanding their groups to different parts of their countries through local nodal representations with a focus based on the requirements in those regions. Similarly, RSG-France organizes workshops annually at their national bioinformatics events.

**Networking**: RSG-France provides informal gatherings through JeBiF Pubs, similarly RSG-Spain’s monthly local meetings to gather the community’s needs and create events.

**Adaptability**: RSG-Bangladesh broadcasts webinar series and workshops in Bangla on YouTube; RSG-France uses the discord channel for communications, while RSR-Turkey uses slack channels and has frequent Instagram broadcasts. RSG-France converted their round table versions to online versions during the pandemic times. RSG-India organizes science writing competitions and webinars relevant to their audience: “Transition to computational biology”, “Career opportunities in computational biology and bioinformatics”, “Intellectual property rights in Bioinformatics”, “Transition to computational biology”, and the much-needed webinar during the pandemic times: “Mental health issues in young researchers”. All the RSGs and the student council are actively recruiting members to expand their teams and associations.

## Sponsorship

We received the amount of 2800 USD from Harvard Medical School. We make the repartition for prizes for eight presenters of each category: three oral presentations (one first place and two second places), three flash talks (one first place and two second places), and two posters (one first place and one second place). On the other hand, the SCS Wikipedia Hackathon 2021 [ 3] took place after the first day of the event, for which 500 USD were allocated in the form of 5 prizes for the winners of this event.

## Fellowships

**Figure 7.**
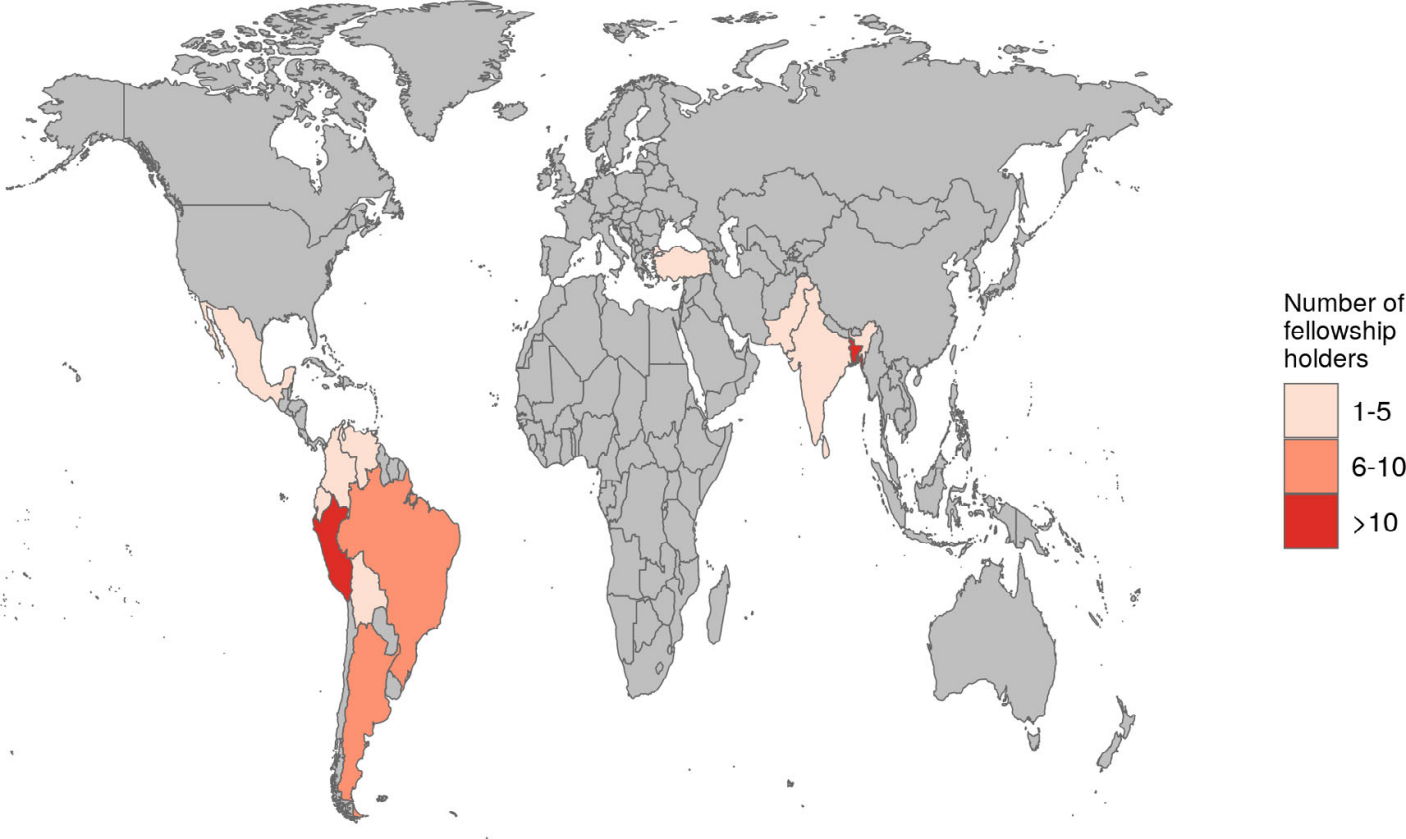
World map distribution of granted fellowships to virtually attend SCS2021. This Figure was created with the ggplot2 R package. ^3^

We provided 73 fellowships for speakers and participants that demonstrated a financial need to attend the event. This fellowship covered the fee for SCS2021 registration, which had an individual cost of 20 USD. The CABANA Project [ 4] sponsorship covered 50 fellowships for Latin American participants (from countries and territories listed in the DAC List of official development assistance Recipients), and the Harvard Medical School [ 5] contribution allowed the participation of 23 people from around the world. Applicants had to send their motivational letters and curriculum vitae for evaluation purposes. Fortunately, thanks to the generous contribution of our sponsors, all the eligible fellowship applicants were awarded. Our fellowship program helped students and professionals with different backgrounds, career stages, ethnicity, and genre, to attend an international event that could potentially boost their careers in Computational Biology. The generous sponsorship of CABANA and Harvard Medical School and the online platform of the event allowed us to provide the highest-ever number of fellowships in the history of the SCS events. Furthermore, fellowships were awarded to people from a wide range of regions, mainly from Latin American and Asian developing countries, as depicted in Figure 7. It is worth noting that due to its virtual nature, fellowships can be covered with much less money than when SC symposia were held in person in the past. Another point to note is that we were able to award all eligible applicants, with a large majority of them coming from the Latin American region. This points out several aspects of our organization: 1) People from Latin America have an economic situation that makes them make an effort to write an application for fellowships even if the cost is only 20 USD. This might be less important for people who attend from the global north, where the impact of the registration fee on the student’s stipends or their laboratories budget is insignificant. 2) Our network and outreach in Latin America are large enough so people can get to know about the SCS events and also the fellowship’s program. 3) Other non-economically favored regions, like some parts of Asia and Africa, are not represented demographically among the fellowship’s applicants. This might be partly due to a lack of presence from our organization in their territory, and hence we are not able to get them involved or reach them through our communication channels. We have taken notes about the latter and are currently dedicating large amounts of effort to strengthen our presence in Asia and Africa.

## Award winners

The SCS2021 awarded eight prizes: three for the best oral presentations, three for the best flash talks, and two for the best poster presentations. The winners were selected by popular selection among the participants, who filled up an online form. The oral presentation prizes were awarded to Thalia Silvestre, Natasha Kitchin, and Ali Al-Fatlawi. Natasha and Ali shared second place. Their presentations were about “The gut microbiota’s influence in the development of Foetal Alcohol Spectrum Disorders” and “Deep Learning Improves Pancreatic Cancer Diagnosis Using RNA- Based Variants”, respectively. Thalia was awarded the best oral presentation prize, and her talk was about “Immunoinformatic selection of potential epitopes in Bordetella pertussis”. The flash talk prizes were awarded to Grace Ko, Jake Crawford, and Cristian Grisales-Vargas. Grace and Jake shared second place. Their presentations were about “A framework to discover the genomic risks of Alzheimer’s disease” and “Prediction of cancer mutation states across multiple data modalities reveals the utility and redundancy of gene expression and DNA methylation”, respectively. Cristian was awarded the best flash talk prize, and her talk was about “Metabiome: a flexible and modular pipeline for metagenomic analysis”.

The poster presentation prizes were awarded to Manuel Moreno-Pérez (1st place poster) and Ina Maria Deutschmann (2nd place poster), and their talks were about “Search for antiviral drugs against SARS-CoV-2 using machine learning methods” and “Disentangling marine microbial association networks”, respectively.

## Discussion

Figure 1 shows that the SCS2021 had attendees from all over the world. However, the participation of students from different regions of the world was not the same. Of all the registered students, 70% came from countries with high-income economies, 20% and 9% were from countries with upper-middle and lower-middle income economies, respectively, and only 1% of the registered participants came from countries with low-income economies. This information put on evidence the high prevalence of attendees from developed countries, although there were a considerable amount of students from developing countries. We promoted the participation of students from developing countries with fellowships ( Figure 7), and the online format of the symposium allowed us to offer the highest number of these awards in the history of SCS events. We afforded these fellowships with sponsorship from CABANA for Latin American students and the Harvard Medical School for students around the world. For future symposia, a strategy to offer more fellowships to help students from developing countries could be to get in contact with further initiatives like CABANA, which has dedicated funding programs to fund people from developing countries. As a result, we could strengthen our networks in these regions (including Africa) to increase the number of people attending our events. Likewise, to know their interest in the Student Council and analyze their needs. We could open access to these events for motivated students with financial barriers and help them to boost their careers in Bioinformatics.

## Online vs offline events

Over the last two years, the COVID-19 pandemic has significantly restricted travel and human contact. Like every other community, the scientific community also had to undergo a drastic transformation, especially with the organization of scientific events. Many events had to be canceled or postponed. Others that did manage to take place had to take place virtually. This led to the introduction of unseen challenges such as 1) the exploration and adoption of the best platform (in terms of accessibility, features that make it close to in-person events, as well as low bandwidth requirements), 2) planning the schedule of the event so everyone, in all time zones, has the best chance to attend most of the event. These challenges were unprecedented as in-person events do not experience them. However, in-person events have their own set of problems. One of the most pressing issues is the significant carbon footprint these international events leave. According to the analysis done by Ref. 10, if each researcher would travel for one scientific event per year, the annual carbon emission of these scientific events would be at least equivalent to that of some small nations. Another major challenge with in-person events is the planning needs. The schedules are easy to manage in these cases. However, the logistics of physical location, meals, and accommodation require months of planning. Lack of equity and inclusivity is also one of the challenges of physical events. Factors like ethnicity, geographical setting, passport and visa requirements, health insurance, and mobility, to name some, strongly influence access to the personal and financial support needed to attend these events. Having a virtual event helps to overcome many of these barriers and allows events to be more accessible and inclusive to the scientific community. ^11^ Apart from the unprecedented challenges of organizing a virtual event, there are also some weaknesses of such events. Most importantly, none of the online meeting platforms have been able to match the level of interaction and networking that would take place in a physical event. ^12^ In many seminars, the participants are not even able to see who the other participants are. Therefore, there is no opportunity for the participants to share ideas or even pleasantries during a coffee break. Some platforms that do allow breakout rooms for social interactions have observed that only a small percentage of participants take part in them. Therefore, having a virtual event has its pros and cons. With more and more scientific events moving to the virtual setting, the platforms hosting these events are spending more time and resources on making these platforms as close to real-life experiences as possible. In this regard, networking becomes one of the most affected dimensions with a special impact on younger attendees and people who are in critical parts of their careers where networking becomes a fundamental part of scientific life, i.e., establishing new collaborations and looking for the next step job opportunities like Ph.D., postdoc, or industry. In order to improve the accessibility of these online events, many are recorded and made available to the participants after their completion via various online platforms. Our symposium is available via the ISCB YouTube channel. This would allow even those participants with low internet bandwidth or with a huge time difference are able to attend all the talks. This would also allow participants to revisit the talks that they found interesting and inspiring. To mitigate the issue of low participation during social sessions, event organizers focus on making these sessions such that people get the opportunity to meet in small groups and perform fun activities. Keeping this in mind, we introduced a social session on each day of our event where participants divided themselves into groups of 8 and played different games that we had planned. These included but were not limited to Kahoot Quiz, Skkribl, and Codenames. We also had an open session for participants to get together for as long as they wanted. It was observed that participants had fun and memorable interactions throughout these sessions. Such methods can be used in future events to ensure that participants are active and have a fun interactive experience.

## Comparison with past events

When comparing previous in-person events like SCS2017 ^1^ and SCS2016, ^13^ we can see that this online SCS2021 version had indeed a better participation rate (approximately 200%). Therefore, by keeping the SCS event online, we could observe more participation from across the globe and hence, helped to improve the event’s inclusivity. A similar trend was observed in SCS2020 ^14^ when SCS was organized online for the first time. SCS2021 observed nearly 100 more participants than its 2020 counterpart, maybe thanks to our social media presence improvement fueled by SCS2020. Another aspect to mention regarding SCS2021 was the use of Airmeet instead of Zoom, which provided significantly more flexibility for the participants to interact with each other as well as the speakers. The virtual tables also gave participants a more realistic feeling of the conference compared to SCS2020. However, Airmeet is not perfect due to its restrictive environment, and we expect new platforms to emerge in the upcoming years to improve the virtual experience of SC events.

In conclusion, the SCS2021 was the first virtual and global flagship event of the ISCB Student Council, which had a better schedule distribution so that all participants around the world had a greater opportunity to attend the presentations. Day 1 was focused on Europe, Africa, and Asia, and Day 2 was focused on being easier to attend to people in the Americas. The inclusion of a round table, the first of its kind within SCS, where various RSGs worldwide presented their progress, discussed different points of view, and reached certain conclusions for the improvement of the community, was a space for great learning. Other activities, such as social events, energized the virtual experience. The platform that was used provided greater interaction between our attendees, allowing for a better experience and interaction opportunities for everyone. We believe that these aspects made it possible for our community to celebrate a truly global event for the ISCB Student Council, even in the middle of the unfortunate pandemic situation. All the details of our event are available at www.scs2021.iscbsc.org.

## Footnotes

^1^

https://www.iscb.org/ismbeccb2021

^2^

https://bioinfo4women.bsc.es/

^3^

https://www.iscb.org/ismbeccb2021-program/hackathon-iscb-wikipedia

^4^

www.cabana.online/

^5^

hms.harvard.edu/
